# Carob-Based Bigel Microstructure Modulates the Gastrointestinal Release of D-Pinitol, Phenolics and Lipids

**DOI:** 10.3390/foods15152744

**Published:** 2026-08-04

**Authors:** Susana Cofrades, Arancha Saiz, Alicia Gutiérrez, María Dolores Álvarez

**Affiliations:** Institute of Food Science, Technology and Nutrition (ICTAN-CSIC), c/José Antonio Novais, 6, 28040 Madrid, Spain; a.saiz@ictan.csic.es (A.S.); a.gutierrez@ictan.csic.es (A.G.)

**Keywords:** hydrogel–oleogel systems, carob extracts, D-pinitol, phenolic compounds, bioaccessibility, gastrointestinal digestion, microstructure, lipid digestion

## Abstract

Carob-based bigels (BGs) were developed as structured fat replacers and delivery systems for bioactive compounds during gastrointestinal digestion. Four formulations combining different hydrogel (HG)/oleogel (OG) ratios and selective incorporation of two carob extracts—an inositol-rich extract (I-CFE) in the HG phase and a phenolic-rich extract (P-CFE) in the OG phase—were evaluated to determine the influence of microstructure on digestive behavior and bioactive release. Confocal microscopy showed that BG structure was mainly governed by the OG network, while alginate-rich aqueous domains remained dispersed and confined to different extents depending on formulation. This organization strongly influenced hydration, enzyme accessibility and matrix breakdown during digestion. D-Pinitol remained stable throughout gastrointestinal digestion, and its release depended on the accessibility of hydrated aqueous domains. Phenolic release and antioxidant activity were also modulated by matrix architecture and extract localization, with BG-70/30-I+P showing the most efficient and sustained antioxidant response. Lipid digestion and fatty acid bioaccessibility were similarly influenced by BG structure: less compact OG networks promoted greater lipase accessibility and higher bioaccessibility of unsaturated fatty acids, whereas more structured lipid matrices partially restricted lipolysis. Overall, BG functionality was governed primarily by microstructural organization rather than composition alone.

## 1. Introduction

The increasing prevalence of metabolic disorders, particularly type 2 diabetes mellitus, has intensified interest in the development of functional foods capable of delivering bioactive compounds with clinically relevant metabolic effects. In this context, structured food systems that simultaneously provide technological functionality and enable the targeted delivery of bioactive compounds have emerged as a promising approach for dietary intervention [1]. Among plant-derived sources of functional ingredients, carob fruit extracts (CFEs) have attracted growing attention due to their richness in bioactive compounds and their availability as sustainable agro-industrial by-products [2,3].

Carob pods are a natural source of inositols, with D-pinitol (present in the I-CFE extract) as the predominant form, accompanied by smaller amounts of myo-inositol. D-pinitol has been widely reported to exert antidiabetic and insulin-sensitizing effects through mechanisms such as improved insulin signaling, reduced hepatic glucose production, and enhanced peripheral glucose uptake [3,4]. Moreover, Pérez-Piñero et al. [5] recently demonstrated that a carob-based liquid concentrate rich in D-pinitol reduced HbA1c and fasting glucose in individuals with prediabetes. Carob pods are also rich in phenolic compounds, particularly highly condensed tannins or non-extractable proanthocyanidins (present in the P-CFE extract), which exhibit antioxidant, anti-inflammatory, and glucose-modulating properties [3]. These compounds have been associated with inhibition of carbohydrate-digesting enzymes, modulation of lipid metabolism, and attenuation of postprandial glycemic responses [6,7]. Due to their high degree of polymerization and limited solubility, condensed tannins behave similarly to insoluble dietary fiber, contributing not only to bioactivity but also to structural reinforcement in food matrices.

Despite their complementary biological functions, the simultaneous incorporation of inositols (I-CFE) and phenolic compounds (P-CFE) into a single food system remains a technological challenge. Their differing polarity, solubility and interaction mechanisms often lead to differences in stability and release kinetics during gastrointestinal digestion [6].

In this context, bigels (BGs)—biphasic systems composed of a hydrophilic hydrogel (HG) and a lipid-structured oleogel (OG)—have emerged as versatile systems for the co-delivery of hydrophilic and hydrophobic bioactives [8,9]. Their dual-phase configuration enables the spatial distribution of compounds according to their physicochemical properties while maintaining mechanical integrity and structural stability. BG microstructure, governed by the nature and relative proportion of each phase, plays a key role in controlling mass transfer processes, enzyme accessibility and the release of bioactive compounds during gastrointestinal digestion [10,11]. Hydrophilic compounds incorporated into the HG phase—such as ascorbic acid, polyphenols or water-soluble vitamins—have been shown to exhibit faster release when aqueous domains are more accessible or less structurally confined, whereas their diffusion can be significantly delayed when entrapped within compact or lipid-dominated matrices [8,10]. Conversely, lipophilic compounds localized within the OG phase—including fat-soluble vitamins, carotenoids and model compounds such as curcumin—depend strongly on lipid matrix breakdown and lipase accessibility for their release, which is in turn influenced by the degree of lipid structuring and network density [9,11]. Studies using β-carotene and curcumin as model lipophilic bioactives—including co-delivery approaches with hydrophilic compounds—have shown that increasing OG content and network compactness can significantly modify gastrointestinal release and bioaccessibility [9,12,13].

Furthermore, the HG/OG ratio has been reported to modulate not only the mechanical properties of BGs but also mass transfer phenomena, enzymatic accessibility and bioactive bioaccessibility during digestion. Structural transitions (ranging from dispersed systems to more interconnected or phase-inverted matrices) have been directly linked to changes in lipid digestion and bioactive release, highlighting the importance of rational microstructural design in these systems, as emphasized in recent reviews on BG-based delivery systems [9,12,14].

In a previous work [15], we characterized the technological properties of carob-based BGs, including their rheological behavior, thermal transitions, physicochemical attributes, microstructure and storage stability. These structural and rheological features are consistent with the behavior typically reported for BGs as composite biphasic systems, where microstructural organization governs macroscopic performance [16]. Our previous study established how the HG/OG ratio and the incorporation of I-CFE and P-CFE affected network formation and mechanical performance, demonstrating their suitability as structured fat replacers. In contrast, the present study addresses a different research question focused on gastrointestinal digestion, bioactive release, antioxidant response and lipid bioaccessibility—functional aspects that were not investigated in our earlier work. These previously characterized structural and rheological features provide the technological foundation required to interpret the digestive behavior evaluated here.

We hypothesize that the degree of confinement of aqueous domains and the organization of the lipid network will determine hydration, enzyme accessibility and matrix breakdown, thereby modulating the release and bioaccessibility of both hydrophilic and lipophilic bioactives during gastrointestinal digestion.

Therefore, the aim of this study was to evaluate how the structural configurations of carob-based BGs incorporating I-CFE and P-CFE influence the release of inositols and phenolic compounds, antioxidant activity and lipid bioaccessibility during in vitro gastrointestinal digestion.

## 2. Materials and Methods

### 2.1. Materials

**Figure 1:**
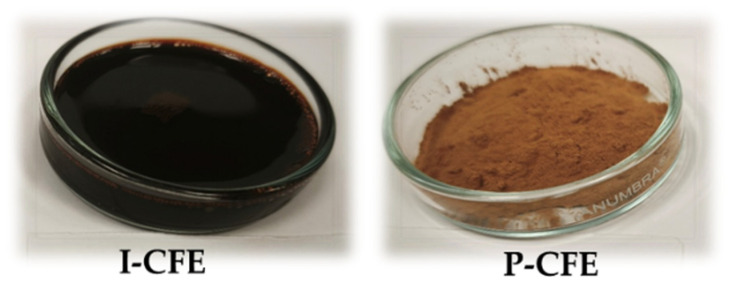
Visual appearance of the two carob fruit extracts (CFEs).

Two carob (*Ceratonia siliqua* L.) fruit extracts (Figure 1) were kindly supplied by PlantTech Biotechnology Spain S.L. (Valencia, Spain). The inositol-rich carob fruit extract (I-CFE; NOW^®^ Energy, Bloomingdale, IL, USA) consisted of a fully soluble fermented carob syrup (290 g/kg of D-pinitol + myo-inositol), in which sugars had been removed during processing. Due to its small-molecule hydrophilic nature, the extract dissolved completely in the HG phase. The phenolic-rich carob fruit extract (P-CFE; NOW^®^ Weight) was supplied as a fine powder and incorporated into the OG phase. The extract contained ≥30% polyphenols (condensed tannins) and ≥70% total dietary fiber (≥65% insoluble fiber, dry matter). These percentages are not independent: the condensed tannins quantified as polyphenols are analytically included within the insoluble dietary fiber fraction according to the manufacturer’s datasheet. These condensed tannins behaved as insoluble, structurally reinforcing components within the OG network. Olive pomace oil (OPO) was kindly donated by the Interprofesional del Aceite de Orujo de Oliva (ORIVA, Seville, Spain) and used as the lipid phase for OG preparation. Microcrystalline wax was also donated by Iberceras (Madrid, Spain) and served as the organogelator. Sodium alginate (Sigma-Aldrich, Steinheim, Germany, analytical grade) was used as the HG matrix. The enzymes used, including pepsin from porcine gastric mucosa (≥2500 U/mg, P7012), pancreatin from porcine pancreas (8 × USP, P7545) and bile extract porcine (B8631), were acquired from Sigma-Aldrich Chemie GmbH (Steinheim, Germany). Fluorophores Nile Red (cat. no. 72485) and Fast Green (cat. no. F7252) were also obtained from Sigma-Aldrich Chemie GmbH (Steinheim, Germany). The Folin–Ciocalteu reagent, Na_2_CO_3_, gallic acid, ascorbic acid, ABTS, and TPTZ were likewise purchased from Sigma-Aldrich (Steinheim, Germany). Supelco 37 FAME Mix (47885-U; Bellefonte, PA, USA) was used as the fatty acid standard. Unless otherwise stated, all reagents were of analytical grade.

### 2.2. Preparation of Bigels (BGs)

#### 2.2.1. Preparation of HG and OG Phases

To prepare 100 g of HG, 98 g of I-CFE were used as the aqueous phase and mixed with 2 g of sodium alginate (SA) under magnetic stirring (400 rpm, 30 min, room temperature) until complete hydration of the polymer. The OG (100 g) was prepared by heating 85.25 g of OPO, 12.5 g of microcrystalline wax (MW), and 2 g of P-CFE at 70 °C until the wax was fully melted and a homogeneous lipid phase was obtained. Subsequently, 0.25 g of soy lecithin was added, and the mixture was homogenized using an Ultra-Turrax (IKA-Werke GmbH & Co. KG., Staufen im Breisgau, Germany) (9000 rpm, 1 min) to ensure complete dispersion of the organogelator and extract.

Control formulations were prepared following the same procedures, with the following adjustments. For the HG, water replaced I-CFE while maintaining 2 g of SA. SA was used without ionic crosslinking to obtain a weakly structured hydrogel phase, ensuring that HG domains remained hydrated and deformable, preventing strong gelation that could dominate the biphasic architecture and interfere with the intended microstructural modulation driven by the HG/OG ratio. For the OG, P-CFE was omitted and the OPO content was adjusted to 87.25 g to preserve the total mass. All phases were used immediately after preparation.

The proportions of SA in the HG (2%) and MW in the OG (12.5%) were selected based on our previous work [15], where these concentrations had been optimized to yield stable and reproducible gel phases.

#### 2.2.2. Preparation of BGs

The preparation of the BGs followed the procedure previously described by our group [15]. As in the cited work, two variables were considered: the hydrogel-to-oleogel (HG/OG) ratio (70/30 and 30/70) and the incorporation of CFEs. Six formulations were prepared and coded according to their composition: BG-70/30-I and BG-30/70-I (containing I-CFE in the HG phase), BG-70/30-I+P and BG-30/70-I+P (containing I-CFE in the HG phase and P-CFE in the OG phase), and BG-70/30 and BG-30/70 as extract-free control systems. Briefly, the OG (at approximately 60 °C) was first homogenized (Ultra-Turrax, 9000 rpm, 30 s). The HG, preheated to the same temperature, was then added, and the mixture was further homogenized (9000 rpm, up to 4 min). The resulting BGs were poured into sterile containers, allowed to stabilize at room temperature, and stored at 4 °C for 72 h prior to analysis. The detailed compositions are provided in Table 1.

Among the evaluated formulations, BG-30/70 systems contained the highest absolute lipid content due to their larger OG fraction, whereas BG-70/30 systems exhibited higher proportions of HG components, establishing the structural contrast that governed all subsequent digestion behaviors.

The complete set of BG formulations, including systems containing only P-CFE, was previously developed and characterized in our earlier work [15]. In the present study, the digestion experiments were intentionally limited to a subset of formulations selected to investigate the effect of BG microstructural organization and the co-delivery of bioactive compounds on gastrointestinal behavior. Therefore, formulations containing only P-CFE were not included. While this choice does not allow the individual release kinetics of P-CFE to be assessed independently, it enables direct comparison between extract-free, I-CFE-loaded, and combined I-CFE/P-CFE systems, which constituted the main objective of this work.

The technological characterization of these BGs (rheology, thermal transitions, physicochemical properties, microstructure and storage stability) was previously reported in our earlier work [15]. In the present study, only the gastrointestinal digestion experiments, CLSM observations, bioactive release analyses, antioxidant assays and lipid bioaccessibility measurements were performed.

### 2.3. Confocal Laser Scanning Microscopy (CLSM)

BG microstructure was examined using a confocal laser scanning microscope (CLSM). A drop of freshly prepared BG (~10 µL) was placed on a glass slide and sequentially stained to visualize the hydrogel and oleogel phases. Fast Green (5 µL, 0.01% in water) was first applied to label the hydrophilic HG domains, using a laser excitation line at 633 nm and an emission range of 650–720 nm (red channel). Subsequently, Nile Red (5 µL, 0.01% in ethanol) was added to stain the lipid-rich OG regions, with excitation at 488 nm and emission between 505–560 nm (green channel). The extremely low amount of ethanol introduced with the dye does not affect the lipid network or alginate domains, and Nile Red prepared in ethanol is a standard staining protocol for CLSM lipid visualization. Images were recorded with a confocal microscope (Leica TCS SP5 AOBS, Mannheim, Germany) equipped with 20× objectives and an optical zoom of up to 5× in selected areas. Laser lines were provided by an Ar laser and a DPSS laser. Detection windows were adjusted to avoid spectral crosstalk between fluorophores. CLSM images were used to assess phase continuity, the size and morphology of HG-rich regions, the spatial distribution of dispersed domains, and structural differences associated with extract localization. All CLSM observations were performed on freshly prepared BGs to ensure that the microstructure reflected the initial phase organization prior to digestion.

### 2.4. In Vitro Gastrointestinal Digestion (GID)

The gastrointestinal digestion behavior of the BGs (BG-70/30-I, BG-30/70-I, BG-70/30-I+P, BG-30/70-I+P, BG-70/30, and BG-30/70) was evaluated under simulated in vitro conditions following the standardized INFOGEST protocol, using the fluid composition and recommendations described by Brodkorb et al. [17]. The complete digestion procedure, including oral, gastric, and intestinal phases, has been previously described in detail [18] and was applied in this study with minor modifications. To ensure comparability among samples, the amount of BG used in each digestion corresponded to 0.5 g of fat.

A preliminary titration was performed using a pH-stat system (TitroMatic 1S, Crison, Alella, Spain) to determine the required NaOH and HCl volumes for pH adjustments. Subsequently, independent digestions were carried out in a shaking incubator (Roto-Therm 2024 Plus, Benchmark Scientific (Sayreville, NJ, USA)) at 37 °C and maintaining continuous agitation throughout the process. Three independent digestion experiments were conducted for each sampling time.

Digesta were collected at specific stages of the GID: after 60 min of the gastric phase (G60) and after 10 min (I10), 60 min (I60), and 90 min (I90) of the intestinal phase. The resulting digesta were centrifuged twice (19,000 rpm, 60 min, 4 °C) to obtain the soluble fraction. Aliquots of these fractions were used for subsequent analyses, including inositol release (sum of myo-inositol and D-pinitol), phenolic compound release, antioxidant activity (ABTS and FRAP), and lipid bioaccessibility.

Blank controls (containing only enzymes and bile) and control BGs (without CFEs) were processed under identical conditions. All digestion experiments were performed in triplicate. For both inositol and phenolic compound determinations, results were corrected by subtracting the values obtained from control BGs to account for background contributions from the matrix.

### 2.5. Determination of Inositol Release

The inositol content of the I-CFE was first quantified using ion-exchange chromatography to determine the initial inositol load incorporated into the BGs. The release of inositols (sum of D-pinitol and myo-inositol) during gastrointestinal digestion was determined in the soluble fractions obtained from the independent digestion assays described above, corresponding to the end of the gastric phase (G60) and to different time points of the intestinal phase: 10 min (I10), 60 min (I60), and 90 min (I90). The release of inositols during GID was quantified using an ion-exchange chromatographic method.

Chromatograms were obtained using a Metrohm (Herisau, Switzerland) ion chromatography system consisting of a Professional IC Vario ONE/SeS equipped with a pulsed amperometric detector (945 Professional Detector Vario), an autosampler (889 IC Sample Center), a Metrosep Carb 2 analytical column (250 × 4 mm i.d., 5 µm particle size), and a Metrosep Carb 2 Guard/4.0 guard column. The flow-through detection cell contained a gold working electrode and an Ag/AgCl reference electrode. Chromatographic data acquisition and processing were performed using MagIC Net software version 4.2.

All analyses were carried out at 35 °C using a mobile phase composed of 250 mM NaOH and 1 mM sodium acetate at a flow rate of 0.5 mL min^−1^. The injection volume was 10 µL and the total run time was 30 min. Appropriate dilutions were prepared with Milli-Q water to adjust myo-inositol and D-pinitol concentrations within the range of the calibration curves.

The results were expressed as the percentage of released inositols (sum of myo-inositol and D-pinitol) relative to their initial content, according to Equation (1):

(1) Release (%) = (C_f_/C_i_) × 100

where C_f_ is the concentration of inositols in the soluble fraction, and C_i_ is the initial inositol content in the BG, theoretically calculated from the inositol content of the I-CFE, considering the amount of extract incorporated into each BG formulation.

### 2.6. Determination of Total Phenolic Release

The total phenolic content of the CFEs was first quantified using the Folin–Ciocalteu method [19] to determine the initial phenolic load incorporated into the BGs. The release of total phenolic compounds during gastrointestinal digestion (gastric and intestinal stages) was then determined in the soluble fractions obtained after centrifugation. Briefly, 25 µL of each soluble fraction were mixed with 25 µL of Folin–Ciocalteu reagent (Sigma-Aldrich, Madrid, Spain). After 5 min, 500 µL of Na_2_CO_3_ solution (75 g/L) and 350 µL of distilled water were added. The mixture was vortexed and incubated for 60 min at room temperature, and the absorbance was measured at 750 nm using a spectrophotometer (Synergy Mx, BioTek Instrument, Winooski, VT, USA). Results were calculated using a gallic acid calibration curve (Sigma-Aldrich, Madrid, Spain) prepared in the corresponding solvent for each fraction and expressed as mg gallic acid equivalents (GAE)/g of BG.

Phenolic release was expressed as a percentage relative to the initial phenolic content, according to Equation (1): where Cf is the concentration of total phenolics in the soluble fraction, and C_i_ is the initial total phenolic content in the BG, calculated theoretically from the total phenolic content of the carob fruit extracts and the amount of extract incorporated into each BG formulation.

### 2.7. Antioxidant Capacity (ABTS and FRAP)

Antioxidant activity (ABTS and FRAP assays) was determined in the I-CFE, P-CFE and in the soluble fractions obtained at the different stages of the GID, namely after 60 min of the gastric phase (G60) and at 10 min (I10), 60 min (I60), and 90 min (I90) of the intestinal phase.

#### 2.7.1. ABTS Radical Cation Scavenging Assay

The ABTS•^+^ radical cation was generated according to Re et al. [20]. Briefly, 38 mg of ABTS were dissolved in 10 mL of 2.45 mM potassium persulfate (Sigma-Aldrich, Madrid, Spain) and the mixture was allowed to react overnight in the dark at room temperature. Before analysis, the ABTS•^+^ solution was diluted with distilled water to an absorbance of 0.80 ± 0.05 at 734 nm. For the assay, 260 µL of the ABTS•^+^ working solution were mixed with 10 µL of sample or the corresponding solvent mixture (blank). The mixture was incubated for 10 min at 30 °C, and absorbance was recorded at 734 nm in a spectrophotometer (Synergy Mx, BioTek Instrument, Winooski, VT, USA). Results were expressed as mg AAE/g BG, and scavenging percentages were interpolated from an ascorbic acid calibration curve used as reference standard.

#### 2.7.2. Ferric Reducing Antioxidant Power (FRAP) Assay

The FRAP assay was carried out according to Benzie and Strain [21]. The FRAP reagent was freshly prepared by mixing 25 mL of acetate buffer (0.3 M, pH 3.6), 2.5 mL of TPTZ solution (10 mM in 40 mM HCl) (Sigma-Aldrich, Madrid, Spain), and 2.5 mL of FeCl_3_·6H_2_O (20 mM) (PanReac AppliChem, Barcelona, Spain). For each measurement, 260 µL of FRAP reagent were mixed with 10 µL of the soluble fraction and incubated at 37 °C for 30 min. Absorbance was recorded at 595 nm using a spectrophotometer (Synergy Mx, BioTek Instrument, Winooski, VT, USA). Results were calculated using an ascorbic acid calibration curve (Sigma-Aldrich, Madrid, Spain) and expressed as mg AAE/g BG.

### 2.8. Fatty Acid (FA) Analysis and Lipid Bioaccessibility

FA profiles of undigested BGs were determined after lyophilization and expressed as mg FAME per g of lyophilized sample, and then converted to mg FAME per g of fresh BG using the moisture content. FA analyses were performed by gas chromatography using an Agilent 7820A gas chromatograph (California, CA, USA). Fatty acids (FAs) were identified by comparing retention times with a commercial standard (Supelco 37 FAME Mix, 47885-U; Bellefonte, PA, USA). For quantification, an internal standard (C13:0) was added to the non-methylated samples prior to methylation.

For lipid bioaccessibility, FAs were analyzed in the soluble fraction obtained after 90 min of intestinal digestion (I90). FA methyl esters were prepared and quantified by gas chromatography following the same analytical procedure described for undigested samples. Lipid bioaccessibility was calculated as the percentage of total FAs detected in the soluble fraction relative to their initial content in the undigested BG.

### 2.9. Statistical Analysis

Statistical analyses were performed using IBM SPSS for Windows, Version 27.0 (IBM Corp., Armonk, NY, USA). All experiments were carried out in triplicate, and results are expressed as mean ± standard deviation. A two-way ANOVA was first conducted to evaluate the effects of formulation, digestion stage, and their interaction. Since a significant interaction was detected for all variables, data were subsequently analyzed using one-way ANOVA to assess (i) the effect of formulation at each stage of the GID and (ii) the effect of digestion stage within each formulation. When significant differences were found, Tukey’s post hoc test (*p* < 0.05) was applied for multiple comparisons. In addition, Pearson correlation coefficients (*r*) were calculated to explore quantitative relationships between selected functional parameters at the end of the intestinal phase (I90), including phenolic release, antioxidant activity (ABTS and FRAP), and fatty acid bioaccessibility. Correlations were considered statistically significant at *p* < 0.05.

## 3. Results

**Figure 2:**
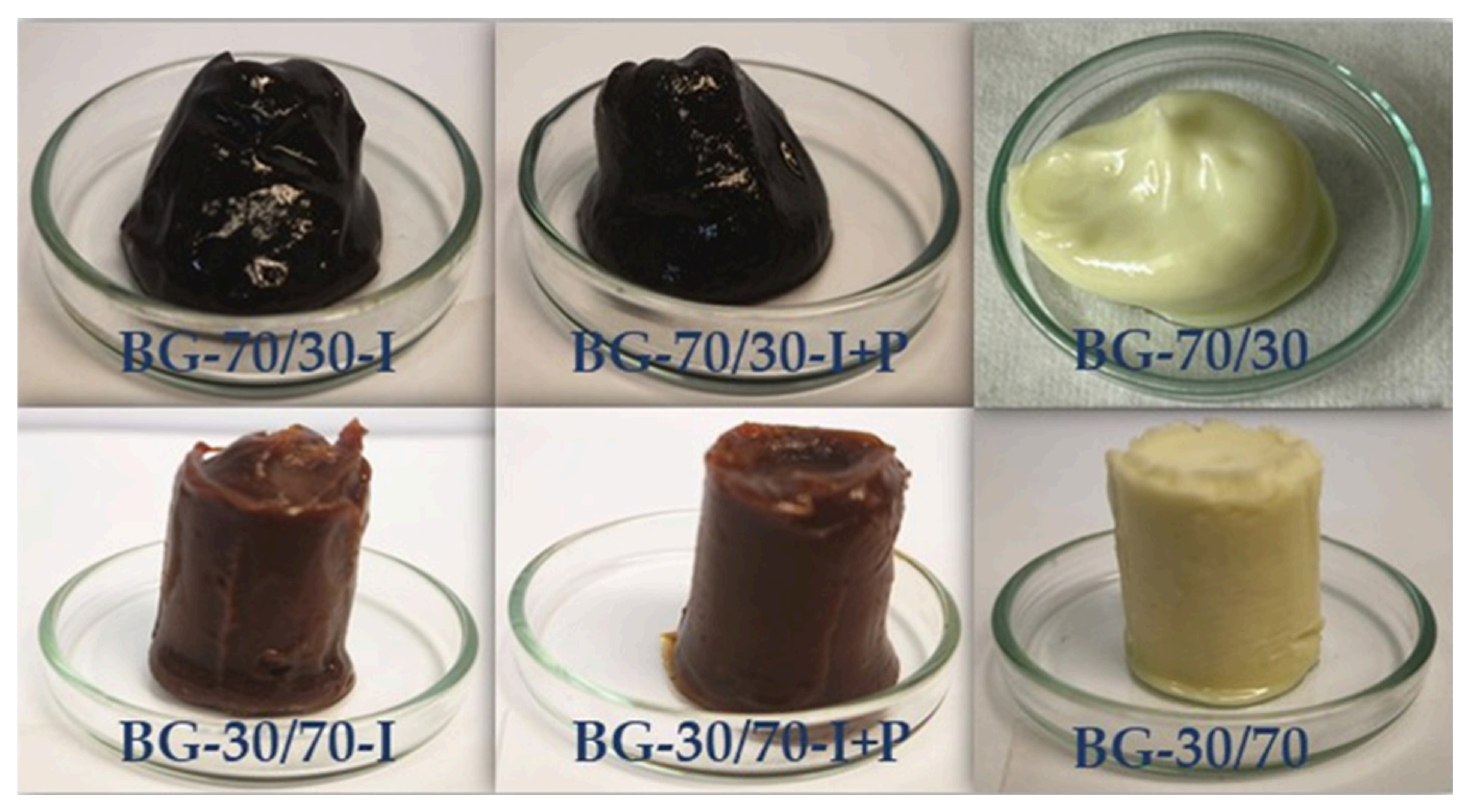
Visual appearance of bigels (BGs) differing in HG/OG ratio (70/30 or 30/70) and extract localization (I-CFE in HG; P-CFE in OG).

Most of the BG formulations investigated in this study were previously developed and characterized in a former work [15], where their rheological, thermal, physicochemical and stability properties were evaluated, together with their microstructural organization using polarized light microscopy. In the present study, extract-free BG-70/30 and BG-30/70 systems were additionally included as structural controls to complement the digestion experiments. Building on those results, Figure 2 presents the macroscopic appearance of the BG formulations, complementing the previously reported rheological, thermal, physicochemical and microstructural characterization of these systems [15].

Building on this previous characterization, the present work focuses on elucidating the relationship between BG microstructure and functional behavior during gastrointestinal digestion (GID). Specifically, confocal laser scanning microscopy was used to obtain a more detailed visualization of phase organization, and an in vitro digestion model was used to investigate the release of inositols and phenolic compounds, antioxidant activity, and lipid bioaccessibility. In this context, the results presented herein should be interpreted as a complementary second step, aimed at understanding how the previously validated technological properties of these BGs translate into controlled bioactive release and functional performance under digestive conditions.

### 3.1. Confocal Microstructural Characterization of BGs

**Figure 3:**
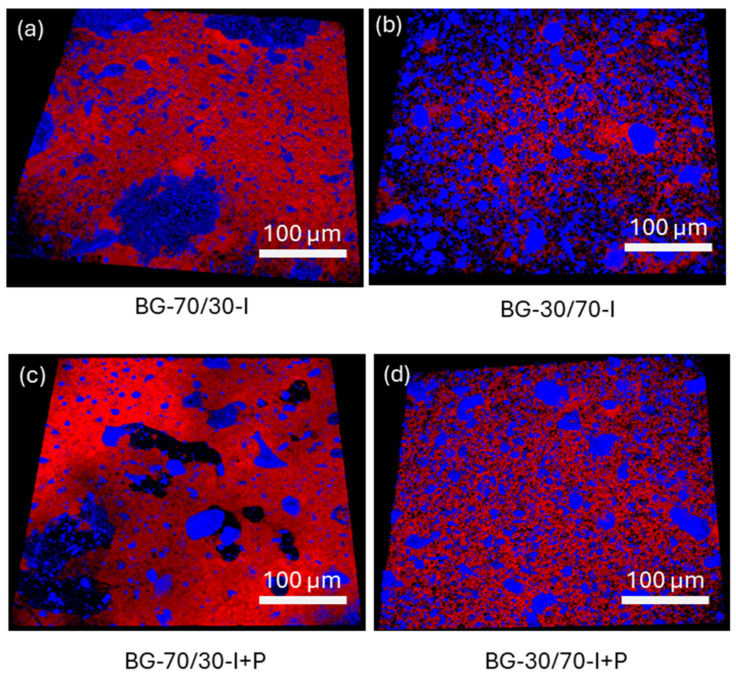
Confocal laser scanning microscopy (CLSM) images of bigels (BGs) with different HG/OG ratios and extract localization. (**a**) BG-70/30-I; (**b**) BG-30/70-I; (**c**) BG-70/30-I+P; (**d**) BG-30/70-I+P. OG phase appears in red and HG phase in blue. Scale bar = 100 µm.

The microstructure of the four BGs with CFEs incorporated was investigated by CLSM to elucidate the effects of the HG/OG ratio and the incorporation of carob extracts on phase distribution, continuity, and matrix organization. The OG phase was selectively stained with Nile Red, which was displayed in red through pseudocolor assignment, whereas the alginate-based aqueous phase (HG), stained with Fast Green, was rendered in blue (Figure 3).

All formulations exhibited a well-defined biphasic microstructure, confirming the successful formation of BG systems, as commonly reported for food-grade BGs structured from hydrophilic polysaccharides and wax-based OGs [14,22]. However, pronounced differences in phase continuity and spatial organization were observed among the samples, indicating that microstructural arrangement was not solely dictated by the nominal HG/OG ratio. In all cases, the OG phase appeared to play a major role in the three-dimensional organization of the matrix.

In BG 70/30 I (HG/OG 70/30, I CFE in HG), CLSM images suggested an extended and interconnected OG network despite the predominance of the aqueous alginate phase in the formulation (Figure 3a). The alginate-rich domains appeared as discrete, highly hydrated inclusions embedded within the lipid matrix rather than forming a continuous structural framework. This microstructural arrangement arises from the absence of ionic crosslinking in the alginate phase, which produced a soft, weakly organized aqueous phase lacking the mechanical integrity required to percolate. These qualitative observations are consistent with the rheological behavior previously reported for BG 70/30 I in our earlier work [15], where the OG phase contributed more strongly to the elastic response and was identified as the main load-bearing component of the network. This behavior aligns with previous reports showing that wax-structured OGs can form mechanically continuous and percolated lipid frameworks even at relatively low lipid fractions [23,24].

In BG-30/70-I (HG/OG 30/70, I-CFE in HG), the OG phase appeared to behave as the continuous matrix, while the alginate-rich aqueous phase was highly fragmented into numerous small domains homogeneously dispersed throughout the lipid network (Figure 3b). This configuration resulted in an increased HG–OG interfacial area and the formation of finely dispersed hydrated domains within the lipid network. Similar microstructures, characterized by finely dispersed aqueous domains within a lipid-rich phase, have been associated with enhanced diffusional pathways and potentially faster release kinetics in biphasic gel systems [25,26], although such relationships must be interpreted with caution when based on qualitative imaging alone.

The incorporation of the P-CFE into the OG phase substantially altered the microstructural organization of the BGs. In BG-70/30-I+P (HG/OG 70/30 with I-CFE in HG and P-CFE in OG), the OG network appeared more compact and heterogeneous than in BG-70/30-I, despite identical HG/OG ratios (Figure 3c). The alginate-rich aqueous domains were larger but more isolated, while regions with reduced fluorescence intensity indicated the presence of more compact lipid domains enriched in P-CFE, consistent with the formation of reinforced OG aggregates. This interpretation was made considering that the localized decrease in fluorescence did not correspond to global quenching or optical attenuation artifacts, as the low dye concentration and the distribution of tannins within the OG phase did not produce diffuse signal loss. The observed patterns were consistent with the microstructural organization previously reported for the same BG systems [15].

Condensed tannins, often regarded as insoluble dietary fiber, have been reported to act as reinforcing fillers in lipid and polymer matrices, increasing network rigidity, tortuosity, and resistance to deformation [27,28].

This structural reinforcement was most evident in BG-30/70-I+P (HG/OG 30/70 with I-CFE in HG and P-CFE in OG), which visually exhibited the most compact and homogeneous microstructure among all formulations, also consistent with the structural features previously described for this formulation in [15]. The OG formed a dense, percolated network that appeared to encapsulate the hydrated alginate phase into small, weakly interconnected domains (Figure 3d). This configuration suggests a high degree of physical confinement of the aqueous alginate domains within a reinforced lipid matrix.

Taken together, CLSM observations indicate that the microstructural organization of the BGs is strongly influenced by the wax-based OG network, which tends to govern phase continuity and the spatial distribution of the alginate phase. The alginate domains, formulated without ionic crosslinking, behaved as soft and highly hydrated compartments whose degree of confinement was determined by the surrounding lipid scaffold. The presence of P-CFE further reinforced the OG network, increasing matrix compactness and enhancing hydrogel confinement. These qualitative findings are in agreement with our previous rheological characterization [15], where formulations with higher OG content and P-CFE incorporation exhibited higher elastic moduli and more solid-like behavior, supporting the role of the OG phase as the main structural backbone of the BGs. This microstructural control over phase continuity and domain confinement is consistent with previous studies demonstrating the key role of BG architecture in governing mass transfer and release behavior during gastrointestinal digestion [22,26,29,30,31,32].

These microstructural differences are not merely descriptive; they have direct implications for digestive behavior. Phase continuity determines the accessibility of digestive fluids, the rate of matrix disintegration and the pathways for mass transfer. Matrices with more accessible or less confined HG domains are expected to facilitate earlier diffusion of hydrophilic compounds and faster penetration of gastric fluids, whereas more compact OG-rich systems may restrict aqueous ingress. In this context, the CLSM observations provide a qualitative mechanistic framework for interpreting the release and bioaccessibility results obtained during in vitro digestion, while recognizing that the microstructural–functional relationships are ultimately supported by the quantitative data presented in the following sections.

### 3.2. Gastrointestinal Digestion and Release Behavior

The in vitro digestion assays were used to evaluate how the structural features of the BGs influenced the release and bioaccessibility of the bioactive compounds present in the CFE. This section examines the release profiles of inositols and phenolic compounds, together with the associated changes in antioxidant activity throughout GID.

#### 3.2.1. Release of Inositols

The release of inositols during simulated gastrointestinal digestion was strongly influenced by the microstructural organization of the BG matrix, particularly the degree of physical confinement of alginate-rich hydrated domains within the lipid network. Inositols such as D-pinitol are cyclitols belonging to the polyol family, characterized by multiple hydroxyl groups and high aqueous solubility, which favor rapid diffusion in hydrated environments. Their behavior is therefore governed primarily by matrix permeability and hydration dynamics, rather than by chemical reactivity.

The quantitative release values obtained during GID are summarized in Table 2, which shows the percentage of inositols (mainly D-pinitol) recovered at each digestion stage for all BG formulations. At the end of the gastric phase (G60), all formulations exhibited high recovery of inositols. The strong hydrophilicity and low molecular weight of D-pinitol facilitate its diffusion once the alginate-rich aqueous domains become hydrated under acidic conditions. In alginate systems, protonation at gastric pH leads to the formation of insoluble alginic acid, which typically induces partial matrix contraction. However, because the alginate phase in these BGs is not ionically crosslinked, it retains a highly porous and hydrated structure that enables rapid diffusion of small hydrophilic molecules. This behavior aligns with observations in plant-based BG systems, where biphasic microstructure and aqueous domain accessibility govern mass transfer and release performance during gastrointestinal digestion [29].

Although inositol release is theoretically expected to follow a non-decreasing trend, slight decreases were observed at intermediate intestinal time points (I60), particularly in BG-30/70-I and BG-30/70-I+P. These fluctuations do not reflect chemical degradation, as values recovered at I90. Instead, they arise from transient physical phenomena inherent to biphasic BGs. During the early intestinal phase, partial reorganization of the OG network and temporary readsorption of hydrophilic compounds into newly formed lipid domains can reduce the amount detected in the aqueous fraction. This effect was more pronounced in formulations containing P-CFE, where the reinforced OG network increases matrix compactness and promotes short-term retention. Additionally, extraction-based quantification may contribute to variability at intermediate time points in OG-rich systems.

BG-70/30-I and BG-70/30-I+P showed the highest gastric release (>83%), consistent with the greater accessibility of their hydrated aqueous domains. Although CLSM revealed that these domains were physically confined within a continuous or co-continuous OG network, such confinement did not prevent the diffusion of small polyols once the matrix became hydrated.

In contrast, BG-30/70-I and BG-30/70-I+P exhibited significantly lower gastric release, reflecting the limited aqueous penetration characteristic of lipid-continuous matrices [1]. The particularly low release observed in BG-30/70-I+P suggests that the incorporation of insoluble fiber and condensed tannins may have contributed to a more structured lipid organization, reducing permeability without affecting the intrinsic chemical stability of D-pinitol. As a cyclitol, D-pinitol is not susceptible to acid hydrolysis or enzymatic degradation under gastrointestinal conditions, and therefore its recovery reflects structural rather than chemical factors.

During the intestinal phase, distinct release kinetics were observed. BG-70/30-I and BG-70/30-I+P maintained relatively stable inositol levels from I10 to I90, consistent with sustained diffusion driven by continued hydration of alginate-rich domains and progressive relaxation of the surrounding lipid network. In contrast, BG-30/70-I showed a marked increase in release at I10 and I90, indicating that its non-reinforced OG network became increasingly permeable as bile salts and lipases disrupted lipid structuring. Because this formulation lacked the phenolic- and fiber-mediated reinforcement present in BG-30/70-I+P, the lipid matrix was more susceptible to enzymatic and surfactant-induced destabilization, facilitating the diffusion of D-pinitol from previously confined aqueous domains.

BG-30/70-I+P consistently showed the lowest inositol release throughout digestion. The more compact lipid organization limited the penetration of digestive fluids and delayed exposure of alginate-rich domains, resulting in slow and incomplete diffusion of D-pinitol. Similar delayed release patterns have been described in structured lipid matrices where phenolic-associated structuring reduces permeability [31]. Systems enriched in insoluble fiber and condensed tannins (BG-30/70-I+P) promoted a delayed and sustained release of D-pinitol, whereas less structured matrices (BG-30/70-I) allowed a rapid intestinal release, reaching almost complete bioaccessibility after 90 min.

Beyond microstructure, potential interactions between alginate, phenolic compounds and D-pinitol may also contribute to the observed release profiles. Alginate can establish hydrogen-bonding interactions with polyphenols, and condensed tannins are known to associate with polysaccharide chains, potentially increasing local viscosity or reducing diffusivity within hydrated domains. Although D-pinitol exhibits weaker affinity for polysaccharides than phenolics, its multiple hydroxyl groups may allow transient, reversible interactions with alginate, which could slightly modulate its mobility in more phenolic-rich systems. These interactions are likely subtle but may complement the structural effects described above.

BG-30/70-I consistently showed the highest inositol bioaccessibility during intestinal digestion, whereas BG-30/70-I+P exhibited the lowest values, highlighting the strong influence of OG continuity and phenolic enrichment on hydrophilic compound release.

From a practical perspective, the sustained release observed in formulations such as BG-70/30-I and BG-30/70-I+P is relevant for applications targeting metabolic health. Prolonged release of D-pinitol may support extended modulation of glycemic response, maintain antioxidant activity over longer digestion times, and enhance the functional potential of BGs designed for controlled delivery of bioactives.

Taken together, the high recovery of inositols across all formulations confirms that the observed differences arise from structural and diffusional factors, not from chemical degradation of the compound. Differences in release among BGs were primarily associated with matrix microstructure, the degree of aqueous domain confinement, and the presence or absence of phenolic- and fiber-associated structural effects.

#### 3.2.2. Release of Total Phenolic Compounds

The release of total phenolic compounds during GID exhibited marked differences among BG formulations, reflecting the combined influence of matrix microstructure, extract localization and physicochemical interactions between phenolics and the surrounding matrix. Phenolic release in structured food systems is strongly influenced by matrix architecture, polarity of the continuous phase and phenolic–matrix interactions [1,6]. The quantitative values corresponding to these trends are presented in Table 3, which summarizes the percentage of total phenolics released at each digestion stage.

During the gastric phase, BG-70/30-I and BG-70/30-I+P exhibited the highest phenolic release. The hydrated nature of alginate-rich aqueous domains facilitated rapid diffusion of hydrophilic phenolics derived from the I-CFE. Under gastric pH, alginate undergoes protonation and forms alginic acid, which may induce partial contraction of the hydrogel phase. Nevertheless, the absence of ionic crosslinking results in a weakly structured and highly porous aqueous matrix that allows efficient diffusion of small hydrophilic compounds. Similar trends have been reported in plant-based bigels, where the organization of aqueous and lipid domains modulates diffusion pathways and influences the release of bioactives during gastrointestinal digestion [29]. BG-70/30-I+P showed slightly higher release than BG-70/30-I, suggesting that phenolics associated with the OG phase (P-CFE) may contribute to early release through interfacial solubilization or weak desorption from the lipid network. Similar early-release mechanisms have been described in phenolic-enriched BGs and OG-based systems [10,31]. Although phenolic release is theoretically expected to increase progressively during digestion, several formulations exhibited non-monotonic profiles, with transient decreases at early intestinal time points (particularly at I10 and I60). These fluctuations do not indicate chemical degradation, as values recovered at I90. Instead, they reflect reversible physical phenomena typical of polyphenols in biphasic matrices. Under intestinal pH, phenolics may undergo temporary precipitation or reduced solubility, followed by re-solubilization as digestion progresses. In addition, weak, reversible interactions with lipid domains or with condensed tannins in P-CFE formulations can promote short-term readsorption, especially in more compact OG networks. Extraction-based quantification may also contribute to variability at intermediate time points in phenolic-rich systems. Together, these factors explain the zigzag-like trends observed in Table 3.

BG-30/70-I showed minimal gastric phenolic release, reflecting the limited aqueous penetration characteristic of lipid-continuous matrices and the absence of phenolic enrichment [1]. BG-30/70-I+P exhibited intermediate release values, indicating that although the more structured lipid organization restricted diffusion from alginate-rich aqueous domains, phenolics associated with the P-CFE phase were able to partially contribute, likely through surface or interfacial interactions. This behavior aligns with reports showing that phenolics located near the lipid interface can be partially released prior to extensive lipid digestion [10,33].

During the intestinal phase, phenolic release profiles diverged more markedly among formulations, particularly during the early stages of intestinal digestion. BG-70/30-I+P exhibited the highest release at I10, showing significantly greater values than the other formulations. This rapid response may be attributed to the combined presence of hydrated alginate-rich domains and dual phenolic localization, which facilitated the diffusion of I-CFE phenolics together with the progressive release of P-CFE-associated compounds as bile salts and lipases destabilized the lipid matrix. Comparable synergistic effects have been reported in biphasic systems where phenolics are distributed between hydrophilic and lipid regions [10,11].

BG-30/70-I+P also exhibited high intestinal release values, although with a more delayed pattern compared with BG-70/30-I+P. The marked increase observed from I10 onwards is consistent with the more compact OG organization identified by CLSM, which likely required partial lipase-mediated disruption before phenolics embedded within or strongly associated with the lipid network became more accessible. Despite the initially slower release, both enriched formulations reached similarly high phenolic release values at I60 and I90, indicating that lipid digestion progressively promoted phenolic liberation regardless of the continuous phase predominance. Similar delayed-release behaviors have previously been described in phenolic-enriched OGs and other structured lipid matrices, where phenolic–lipid associations modulate release kinetics during digestion [31,33].

BG-70/30-I exhibited a transient decrease at I10 followed by a gradual increase throughout the intestinal stage. This behavior may be attributed to the rapid gastric release of readily accessible phenolics from hydrated alginate-rich regions, followed by changes in diffusion dynamics under intestinal conditions and progressive matrix relaxation as digestion advanced. Conversely, BG-30/70-I consistently presented lower phenolic release during most digestion stages, although no significant differences between the non-enriched formulations were observed at I90, suggesting that intestinal digestion eventually promoted a similar extent of phenolic release in both systems.

Beyond structural effects, phenolic–matrix interactions may also contribute to the observed release behavior. Condensed tannins can associate with alginate chains through hydrogen bonding and hydrophobic interactions, potentially increasing local viscosity or reducing diffusivity within hydrated domains. These interactions may be particularly relevant in phenolic-enriched systems, complementing the microstructural effects described above.

From a practical perspective, the enhanced and sustained phenolic release observed in enriched formulations is relevant for functional food applications. Prolonged phenolic availability may support extended antioxidant activity, modulate oxidative stress during digestion, and improve the functional performance of BGs designed for controlled polyphenol delivery.

These findings indicate that phenolic release during gastrointestinal digestion was primarily governed by the interplay between matrix microstructure and extract localization. Phenolic enrichment significantly enhanced the extent of release, particularly during the early intestinal stages, while prolonged digestion reduced the differences between formulations with distinct continuous phases. These findings highlight the importance of multiphase structuring strategies for modulating polyphenol release kinetics in biphasic gel systems [10].

Phenolic-enriched formulations (BG-70/30-I+P and BG-30/70-I+P) showed markedly higher phenolic release—especially at I10—highlighting the strong impact of extract localization and OG microstructure on early intestinal bioaccessibility.

#### 3.2.3. Antioxidant Activity During GID

The evolution of antioxidant activity during gastrointestinal digestion reflected the combined influence of BG microstructure, phenolic localization and progressive matrix disintegration. Both ABTS radical-scavenging activity and FRAP reducing power exhibited formulation-dependent trends that closely mirrored the release of total phenolic compounds, consistent with the well-established relationship between phenolic bioaccessibility and antioxidant response in structured food systems [6,34,35]. The INFOGEST protocol used in this study provides a reliable framework for interpreting changes in antioxidant capacity during digestion [36]. Although phenolics are expected to be the main contributors to antioxidant activity, other compounds present in the digesta—such as inositols, minor reducing sugars, or small organic acids—may also contribute modestly to ABTS or FRAP responses, particularly under intestinal conditions.

During the gastric phase (G60), distinct antioxidant patterns were observed depending on the analytical method employed. In the ABTS assay (Table 4), BG-30/70-I+P showed the highest radical-scavenging activity, indicating an important contribution of phenolics associated with the OG phase. In contrast, FRAP results (Table 5) showed that BG-70/30-I and BG-70/30-I+P exhibited the highest reducing power, with no significant differences between them. This behavior suggests that phenolic localization and matrix organization influenced radical-scavenging activity and reducing power differently during gastric digestion. The relatively high antioxidant activity observed in formulations containing accessible aqueous domains may be attributed to the rapid hydration of alginate-rich regions, which facilitated the early diffusion of hydrophilic phenolic compounds originating from the I-CFE. Similar hydration-driven diffusion behavior has been reported in plant-based BG systems, where the organization and accessibility of aqueous domains modulate the mobility of small hydrophilic compounds during gastrointestinal digestion [29]. Moreover, the elevated ABTS activity observed in BG 30/70 I+P suggests that phenolics associated with the OG phase (P-CFE) also contributed to antioxidant activity during the gastric stage, likely through interfacial localization and partial solubilization. Comparable early-release behaviors have been reported in phenolic-enriched lipid and BG systems [10,31].

Phenolic-enriched formulations (BG-70/30-I+P and BG-30/70-I+P) exhibited the strongest ABTS activity throughout digestion, particularly at I60 and I90, confirming the close correspondence between phenolic bioaccessibility and antioxidant performance.

FRAP values showed more moderate differences among formulations, but phenolic-enriched systems still displayed consistently higher reducing power than non-enriched BGs, especially during the gastric phase.

In contrast, BG-30/70-I showed very limited antioxidant activity during the gastric phase, in agreement with its low phenolic release and the strong physical confinement of alginate-rich aqueous domains within a lipid-dominated matrix lacking phenolic enrichment. BG-30/70-I+P exhibited intermediate-to-high antioxidant values depending on the assay, suggesting that despite the more structured lipid organization, phenolics associated with the P-CFE phase were partially accessible even before extensive lipid digestion occurred. These observations agree with previous studies indicating that antioxidant activity during digestion is primarily governed by the fraction of polyphenols that becomes bioaccessible rather than by the total phenolic content initially quantified, since digestion may release non-extractable polyphenols with relevant antioxidant potential [7,35].

Marked differences among formulations became evident during the intestinal phase. In the ABTS assay, BG-70/30-I+P showed the highest antioxidant activity throughout intestinal digestion (Table 4), with a sharp increase already observed at I10. BG-30/70-I+P also exhibited high ABTS values, although significantly lower than BG-70/30-I+P during the later intestinal stages. In contrast, FRAP responses (Table 5) showed comparatively smaller variations among formulations during digestion. Although BG-70/30-I+P generally presented the highest reducing power values, differences with BG-70/30-I were not always statistically significant.

This behavior reflects the synergistic effect between hydrated alginate-rich aqueous domains and the dual localization of phenolics in both phases. Hydration and relaxation of the aqueous regions, together with the early action of bile salts, promoted rapid solubilization of I-CFE-derived phenolics, while progressive destabilization of the lipid network facilitated the release of P-CFE-associated phenolics. Similar behaviors have been reported in biphasic systems where aqueous accessibility accelerates antioxidant release and lipid disintegration sustains their availability [10,11,37]. BG-30/70-I+P exhibited sustained antioxidant activity throughout intestinal digestion, particularly in the ABTS assay, consistent with the delayed release of phenolics from the more compact OG organization. This pattern agrees with the OG structural features observed by CLSM, which likely required partial lipase-mediated disruption before phenolics embedded in or strongly associated with the lipid matrix became more bioaccessible. Such delayed yet sustained antioxidant responses have previously been described in phenolic-enriched OGs and structured lipid matrices, where phenolic–lipid associations modulate the timing of antioxidant availability [33,38].

BG-70/30-I exhibited a transient decrease at I10 followed by a progressive increase throughout the intestinal stage. This behavior may be associated with the rapid gastric release of readily accessible phenolics from hydrated alginate-rich domains, followed by changes in diffusion dynamics under early intestinal conditions and subsequent matrix relaxation as digestion progressed. As bile salt concentration and ionic strength increased, phenolic diffusion may have been progressively facilitated, leading to the gradual recovery of antioxidant activity [39]. BG-30/70-I consistently remained the formulation with the lowest antioxidant activity throughout digestion. Its lipid-dominated structure, combined with the absence of P-CFE, restricted phenolic release from confined aqueous domains and limited antioxidant contribution even as lipid digestion advanced. This result highlights the limited effectiveness of non-enriched OG matrices for the delivery of hydrophilic antioxidants [1,40].

The comparison between ABTS and FRAP assays provided additional mechanistic insight. ABTS detects radical-scavenging capacity through both hydrogen-atom transfer and single-electron transfer mechanisms and is sensitive to a wide range of hydrophilic and lipophilic antioxidants. In contrast, FRAP exclusively measures ferric-reducing power under acidic conditions and relies solely on single-electron transfer reactions. As a result, FRAP is more selective for compounds with strong reducing potential, whereas ABTS captures a broader spectrum of antioxidant species. These methodological differences explain why ABTS showed sharper increases during intestinal digestion—particularly in phenolic-enriched formulations—while FRAP exhibited more moderate changes.

It is important to note that neither ABTS (Table 4) nor FRAP (Table 5) necessarily follow the same trend as total phenolic release (Table 3). The Folin–Ciocalteu assay quantifies all reducing species, including phenolics of low antioxidant potency and other extract components, whereas ABTS and FRAP are dominated by the most reactive compounds. As a result, increases in total phenolics may coincide with temporary decreases in ABTS or FRAP activity if highly active phenolics undergo transient precipitation, readsorption, or matrix association, as observed in BG-30/70-I and BG-30/70-I+P. Conversely, ABTS or FRAP may increase even when total phenolics decrease, particularly in phenolic-enriched systems such as BG-70/30-I+P, where the release of highly reactive tannins and flavanols enhances antioxidant activity despite fluctuations in total phenolic content. These differences reflect the distinct chemical sensitivities of the assays and the complex redistribution of phenolics during digestion.

A closer alignment between the release of total phenolic compounds and the evolution of antioxidant activity during digestion was observed mainly in the ABTS assay. In contrast, FRAP responses did not follow the same trend and appeared more dependent on the specific redox properties of the phenolic compounds released at each stage of digestion, as well as on additional factors influencing the reducing environment. In addition to phenolics, minor reducing species released during digestion may have contributed slightly to ABTS or FRAP values, although their impact was comparatively small. Formulations exhibiting greater phenolic bioaccessibility generally showed stronger antioxidant responses, particularly in ABTS measurements, whereas lipid-dominated matrices with limited phenolic release displayed lower antioxidant activity. The delayed yet sustained antioxidant response of BG-30/70-I+P mirrored its gradual phenolic release from the more compact OG organization. Collectively, these findings confirm that antioxidant activity in BG systems is primarily governed by phenolic accessibility and release kinetics, in agreement with previous observations in structured food matrices [6,10,34,35,37].

### 3.3. FA Profile of BGs and Bioaccessible Lipid Fraction After Intestinal Digestion

#### 3.3.1. FA Profile of Undigested BGs

The FA composition of the undigested BGs reflected the OPO content of each formulation. As expected, BG-70/30-I/BG-70/30-I+P (70/30 HG/OG) and BG-30/70-I/BG-30/70-I+P (30/70 HG/OG) exhibited identical qualitative and quantitative FA profiles within each pair, due to the use of the same lipid source and processing conditions (Table 6).

In all formulations, monounsaturated fatty acids (MUFA) represented the predominant lipid fraction, with oleic acid (C18:1n9c) as the major fatty acid, followed by palmitic acid (C16:0) as the primary saturated fatty acid (SFA), and linoleic acid (C18:2n6c) as the main polyunsaturated fatty acid (PUFA). These distributions are consistent with the typical FA composition reported for olive-derived oils [41]. Although the absolute FA concentrations differed according to total oil content, the relative proportions of SFA, MUFA and PUFA remained comparable among formulations, indicating that the observed differences during digestion were mainly associated with BG microstructure and lipid matrix organization rather than with intrinsic differences in lipid composition.

The BG-30/70 formulations exhibited substantially higher absolute concentrations of all FA classes due to their larger OG fraction. However, this higher lipid content does not necessarily imply greater lipid bioaccessibility, since lipid release during digestion may be strongly restricted by the more structured lipid organization and enzyme accessibility [1,33]. Therefore, characterization of the soluble fraction after digestion is essential to evaluate the actual extent of FA release and micellar incorporation.

The proportional distribution of FA classes in the undigested BGs showed a predominance of MUFA (approximately 73–75%), followed by SFA (approximately 15–16%) and PUFA (approximately 10–11%), which is characteristic of olive-derived lipid systems. This compositional uniformity provides an appropriate baseline for interpreting differences in lipolysis and FA bioaccessibility as a function of BG microstructure.

BG-30/70 formulations showed markedly higher absolute concentrations of all FA classes due to their larger OG fraction, while the proportional distribution of SFA, MUFA and PUFA remained comparable across formulations, confirming that differences observed during digestion arise from microstructural effects rather than intrinsic lipid composition.

#### 3.3.2. FA Profile of the Soluble Lipid Fraction After Intestinal Digestion

The FA composition of the soluble fraction recovered after intestinal digestion (I90) revealed pronounced formulation-dependent differences, reflecting the influence of lipid matrix architecture, enzyme accessibility to the OG network and potential phenolic–lipid interactions. The quantitative values corresponding to the soluble fraction are presented in Table 7.

BG-30/70 formulations released the highest absolute amounts of all FA classes into the soluble fraction, whereas phenolic enrichment (BG-70/30-I+P and BG-30/70-I+P) consistently reduced MUFA and PUFA release, highlighting the strong influence of OG continuity and phenolic-associated structuring on lipid mobilization during digestion.

All formulations showed substantial release of MUFA into the soluble fraction, particularly oleic acid (C18:1n9c), which remained the predominant FA after digestion. Palmitic acid (C16:0) and linoleic acid (C18:2n6c) also represented important fractions of the bioaccessible lipids. However, the extent of release differed markedly depending on BG composition and structure.

The BG-30/70 systems exhibited the highest absolute concentrations of FAs in the soluble fraction, mainly due to their greater oil content. In particular, BG-30/70-I showed the highest concentrations of SFA, MUFA and PUFA after digestion (Table 7). Nevertheless, these absolute values should be interpreted considering the higher initial lipid content of these formulations. For this reason, FA bioaccessibility percentages were calculated to evaluate the efficiency of lipid release and micellar incorporation independently of the initial FA concentration.

In the BG-70/30 formulations, CLSM micrographs previously revealed a less compact and less continuous OG organization, with hydrated alginate-rich regions distributed throughout the structure. This matrix arrangement likely facilitated enzyme penetration and lipid mobilization during digestion, promoting efficient lipolysis and release of FAs into the soluble fraction. Similar behavior has been reported in biphasic and weakly structured lipid systems where discontinuous lipid networks improve lipase accessibility [10,11].

The incorporation of P-CFE influenced the release behavior differently depending on BG structure. In BG-70/30-I+P, FA concentrations in the soluble fraction were slightly lower than in BG-70/30-I for most FAs, suggesting that phenolic compounds and associated fiber may have partially restricted lipid mobility or modified interfacial accessibility during digestion, although this effect cannot be considered demonstrated and should be interpreted cautiously. A more pronounced effect was observed in BG-30/70-I+P, where the incorporation of P-CFE reduced the concentration of bioaccessible MUFA and PUFA compared with BG-30/70-I. This behavior is consistent with a more structured OG organization associated with phenolic–lipid and fiber–lipid interactions, which may contribute to limiting lipase penetration and delaying lipid digestion, although these interactions were inferred rather than directly measured.

These structural effects became more evident when FA bioaccessibility percentages were considered (Table 8). Among the evaluated fatty acids, PUFA exhibited the highest bioaccessibility values, particularly linoleic acid (C18:2n-6), which reached approximately 56–61% depending on the formulation. MUFA showed intermediate bioaccessibility, with vaccenic and oleic acids generally ranging between 45 and 54%. In contrast, SFA displayed the lowest bioaccessibility values, especially in BG-70/30-I+P, where palmitic and stearic acids reached values close to 25%. The lower bioaccessibility of SFA compared with MUFA and PUFA is consistent with the preferential incorporation of unsaturated FAs into mixed micelles during intestinal digestion, whereas long-chain SFA are more prone to forming insoluble calcium soaps or remaining associated with poorly digestible lipid structures [1,42].

Regarding SFA, higher OG levels provide more accessible triglycerides for lipase action and generate more micellar components, which enhances SFA solubilization. In contrast, 70/30 BGs restrict lipid accessibility, resulting in lower SFA bioaccessibility.

However, MUFA and PUFA do not show the same increase in bioaccessibility than SFA because they are inherently easier to solubilize during intestinal digestion. Their unsaturated structure allows them to incorporate into mixed micelles more efficiently and with less dependence on OG content. In contrast, SFA are poorly soluble and strongly depend on the amount of lipolysis products generated by the OG phase. Therefore, increasing OG mainly boosts SFA bioaccessibility, while MUFA and PUFA remain relatively stable across formulations.

For MUFA, vaccenic acid did not show significant differences among formulations. In contrast, oleic acid exhibited a clear and significant pattern: BG-30/70-I presented the highest bioaccessibility, whereas BG-30/70-I+P showed the lowest. Although phenolic enrichment reduced oleic acid bioaccessibility in both matrices, this reduction was statistically significant only in BG-30/70-I+P. These observations are consistent with earlier studies describing how phenolic-associated structural reinforcement can modulate lipase accessibility in structured lipid systems [33,40].

No significant differences were detected for PUFA, as both linoleic and linolenic acids showed similar bioaccessibility across all formulations. This stability aligns with the well-documented tendency of unsaturated fatty acids to be efficiently incorporated into mixed micelles during digestion, regardless of moderate structural variations in the lipid matrix [1,42].

Overall, the significant differences observed for SFA and oleic acid highlight the influence of matrix organization and phenolic enrichment on lipase accessibility and lipid release, whereas PUFA and vaccenic acid remained unaffected by structural differences among formulations. These findings indicate that the impact of phenolics on lipid digestion is structure-dependent, becoming more evident in matrices with higher OG content.

### 3.4. Linking Microstructure, Bioactive Release, Antioxidant Response and Lipid Bioaccessibility

The results obtained from the microstructural characterization, bioactive release, antioxidant activity and lipid bioaccessibility analyses revealed a coherent mechanistic relationship between BG structure and digestive behavior. Overall, the functional performance of the BG systems during GID was governed primarily by the organization and continuity of the OG network, which controlled both the accessibility of digestive fluids and the mobility of hydrophilic and lipophilic compounds within the matrix. These interpretations are based on CLSM observations and on previously reported structural behavior of wax-based oleogels in biphasic delivery systems.

CLSM observations demonstrated that the wax-based OG acted as the structurally dominant phase in all formulations, determining the degree of confinement of the alginate-rich aqueous domains and modulating the accessibility of digestive enzymes to the lipid phase. Consequently, differences in digestibility and bioactive release were more closely associated with matrix architecture than with differences in FA composition, since all BGs exhibited comparable qualitative lipid profiles dominated by MUFA, particularly oleic acid.

In the BG-70/30 systems (BG-70/30-I and BG-70/30-I+P), the OG organization appeared less compact and more heterogeneous, generating a more open structure. This arrangement facilitated rapid hydration of the alginate-rich regions during digestion and promoted the early diffusion of hydrophilic bioactives, including D-pinitol and I-CFE-derived phenolic compounds. As a result, these formulations showed high phenolic release and marked antioxidant responses, particularly in the ABTS assay, reflecting efficient solubilization and sustained accessibility of antioxidant compounds throughout digestion. The enhanced performance of BG-70/30-I+P further suggests that phenolics associated with the lipid phase became progressively available as digestion advanced, consistent with observations in multiphase phenolic delivery systems.

This structural openness also favored lipase access to the lipid phase, resulting in high FA bioaccessibility (Table 8) and efficient incorporation of MUFA and PUFA into the soluble fraction. These behaviors align with previous reports showing that discontinuous lipid networks promote lipase accessibility in structured lipid matrices [10,11].

In contrast, the BG-30/70 systems (BG-30/70-I and BG-30/70-I+P) exhibited a denser and more continuous OG organization that imposed stronger physical confinement on the alginate-rich aqueous domains. This compact lipid arrangement restricted aqueous penetration during the gastric phase and delayed the release of hydrophilic bioactives, leading to lower phenolic accessibility and reduced antioxidant activity during the early stages of digestion.

The effect became more pronounced after incorporation of P-CFE in BG-30/70-I+P. Phenolic compounds and associated fiber likely contributed to a more structured lipid organization, as suggested by CLSM observations and by the literature describing phenolic–lipid associations in OG systems [33,40]. As a result, both hydrophilic and lipophilic compounds remained partially entrapped within the lipid-continuous matrix until more advanced stages of intestinal digestion. This delayed matrix disintegration explains the progressive phenolic release and sustained antioxidant response observed during the intestinal phase.

The same structural effects also influenced lipid digestion and FA bioaccessibility. Although the BG-30/70 systems contained higher absolute amounts of FAs due to their larger oil fraction, incorporation of P-CFE reduced the bioaccessibility of several major FAs, particularly oleic and linolenic acids. This finding supports the hypothesis that phenolic enrichment influenced lipid organization and limited lipase accessibility during digestion. In all formulations, unsaturated fatty acids exhibited higher bioaccessibility than SFA, consistent with the preferential incorporation of MUFA and PUFA into mixed micelles during intestinal digestion.

To strengthen these mechanistic interpretations, Pearson correlation coefficients were calculated between key functional and digestive parameters (Table 9). Phenolic release showed a strong positive correlation with ABTS antioxidant activity (*r* = 0.909, *p* < 0.001), confirming that the enhanced antioxidant response in the BG-70/30 systems was directly associated with the greater accessibility of phenolic compounds. Inositol release also correlated positively with oleic bioaccessibility (*r* = 0.913, *p* < 0.001), indicating that more open OG structures facilitated both hydrophilic diffusion and lipid digestion. Conversely, antioxidant activity (ABTS and FRAP) exhibited significant negative correlations with total FA bioaccessibility (*r* = –0.716 and *r* = –0.711, respectively; *p* < 0.01), supporting the observation that compact lipid-continuous matrices restricted both lipase accessibility and the release of antioxidant compounds. Additionally, oleic and linolenic bioaccessibility were strongly correlated (*r* = 0.775, *p* < 0.01), reflecting the parallel response of representative unsaturated FAs to differences in OG continuity. Together, these quantitative relationships reinforce the central role of matrix architecture in governing bioactive release, antioxidant performance and lipid digestibility during GID.

Taken together, these results demonstrate that the digestive behavior of BG systems is governed by the interplay between OG continuity, aqueous-domain accessibility and phenolic localization. Less compact and more permeable OG structures facilitated rapid bioactive diffusion, strong antioxidant responses and efficient lipid digestion, whereas more structured lipid-continuous matrices delayed bioactive release and restricted FA bioaccessibility. Furthermore, the strategic localization of phenolics within both aqueous and lipid regions emerged as an important factor modulating not only antioxidant performance but also lipid digestibility and release kinetics.

This integrated understanding highlights the potential of BG microstructure as a design tool for tailoring bioactive delivery, antioxidant functionality and lipid bioaccessibility in structured food systems.

## 4. Conclusions

This study demonstrates that the digestive behavior and functional performance of carob-based BGs were primarily governed by their microstructural organization. Confocal microscopy confirmed that all formulations were structured around a continuous or co-continuous OG network, which controlled the confinement of alginate-rich aqueous domains and modulated water penetration, enzyme accessibility and matrix breakdown during gastrointestinal digestion. These observations provide mechanistic insight into how biphasic architectures govern mass transfer phenomena in structured food systems.

The release of hydrophilic bioactives was strongly influenced by the accessibility of hydrated aqueous regions. Less compact systems, such as BG-70/30-I and BG-70/30-I+P, promoted rapid diffusion of D-pinitol and phenolic compounds, whereas more structured lipid-continuous networks delayed release and promoted a more sustained intestinal availability. Phenolic bioaccessibility and antioxidant activity did not always follow identical trends, as ABTS and FRAP respond selectively to the most reactive phenolic species rather than to total phenolic content. Nonetheless, BG-70/30-I+P exhibited the strongest and most sustained antioxidant activity, reflecting the high bioaccessibility of highly reactive phenolics and the progressive destabilization of the lipid network during digestion. This work therefore identifies specific microstructural features—such as aqueous domain accessibility and phenolic distribution—that can be strategically modulated to tailor release kinetics of hydrophilic antioxidants.

Lipid digestion and fatty acid bioaccessibility were also modulated by BG microstructure. Less compact OG networks facilitated lipase accessibility, lipid hydrolysis and the micellar incorporation of FAs, whereas more structured lipid matrices partially restricted lipid mobilization and delayed digestion. Unsaturated FAs generally exhibited higher bioaccessibility than SFA, consistent with their preferential incorporation into mixed micelles during intestinal digestion. The incorporation of phenolic extracts exerted a structure-dependent effect, slightly reducing FA bioaccessibility in more structured lipid networks while having a less pronounced impact in more accessible matrices. These results highlight how phenolic-associated structuring can influence lipid digestibility, offering a potential strategy for modulating lipolysis in functional fat-replacer systems.

Overall, this study advances the understanding of structure–function relationships in BG systems by demonstrating how microstructural design governs the simultaneous delivery of hydrophilic and lipophilic bioactives.

## Figures and Tables

**Table 1:** Composition of BG formulations (% *w*/*w*) according to the HG/OG ratio.

	HG Ingredients			OG Ingredients			
Bigel (BG)	I-CFE	Water	SA	P-CFE	OPO	MW	Soy Lecithin
BG-70/30-I	68.60	0.00	1.40	0.00	26.18	3.75	0.08
BG-30/70-I	29.40	0.00	0.60	0.00	61.08	8.75	0.18
BG-70/30-I+P	68.60	0.00	1.40	0.60	25.58	3.75	0.08
BG-30/70-I+P	29.40	0.00	0.60	1.40	59.68	8.75	0.18
BG-70/30	0.00	68.60	1.40	0.00	26.18	3.75	0.08
BG-30/70	0.00	29.40	0.60	0.00	61.08	8.75	0.18

BG-70/30-I and BG-30/70-I: bigels containing I-CFE in the HG phase; BG-70/30-I+P and BG-30/70-I+P: bigels containing I-CFE in the HG phase and P-CFE in the OG phase; BG-70/30 and BG-30/70: extract-free control systems; I-CFE, inositol-rich carob fruit extract; P-CFE, phenolic-rich carob fruit extract; SA, sodium alginate; OPO, olive pomace oil; MW, microcrystalline wax.

**Table 2:** Inositol release (%) during simulated gastrointestinal digestion (GID) in BG formulations.

Inositols (%)	BG-70/30-I	BG-30/70-I	BG-70/30-I+P	BG-30/70-I+P
G60	84.80 ± 3.47 Aa	77.48 ± 3.15 Bb	83.03 ± 0.35 A,Ba,b	67.90 ± 3.46 Cb
I10	86.36 ± 2.24 Ba	94.48 ± 1.58 Aa	84.43 ± 1.09 Ba	83.04 ± 0.56 Ba
I60	84.93 ± 2.34 Aa	84.64 ± 5.55 Ab	82.75 ± 1.70 Aa,b	72.04 ± 1.81 Bb
I90	88.22 ± 1.70 Ba	96.74 ± 2.75 Aa	80.51 ± 1.61 Cb	72.83 ± 2.40 Db

G60: 60 min gastric phase. I10, I60, I90: 10, 60 and 90 min intestinal phases, respectively. Mean values (*n* = 3) ± standard deviation. Different uppercase letters within rows indicate significant differences among formulations (*p* < 0.05). Different lowercase letters within columns indicate significant differences among digestion stages (*p* < 0.05). For sample denominations, see Table 1.

**Table 3:** Phenolic release (%) during gastrointestinal digestion (GID) in BG formulations.

Total Phenolics (%)	BG-70/30-I	BG-30/70-I	BG-70/30-I+P	BG-30/70-I+P
G60	48.25 ± 0.46 Ab	11.54 ± 1.28 Cc	51.20 ± 0.65 Ac	32.00 ± 2.75 Bb
I10	32.32 ± 0.85 Cd	36.83 ± 2.65 Cb	82.49 ± 1.88 Aa	74.87 ± 4.46 Ba
I60	42.64 ± 0.91 Cc	56.93 ± 1.92 Ba	72.41 ± 1.55 Ab	75.59 ± 2.89 Aa
I90	54.82 ± 2.59 Ba	58.52 ± 3.84 Ba	78.63 ± 1.83 Aa	74.52 ± 1.39 Aa

G60: 60 min gastric phase. I10, I60, I90: 10, 60 and 90 min intestinal phases, respectively. Mean values (*n* = 3) ± standard deviation. Different uppercase letters within rows indicate significant differences among formulations (*p* < 0.05). Different lowercase letters within columns indicate significant differences among digestion stages (*p* < 0.05). For sample denominations, see Table 1.

**Table 4:** ABTS radical-scavenging activity (mg AAE/g BG) during simulated gastrointestinal digestion (GID) in BG formulations.

ABTS	BG-70/30-I	BG-30/70-I	BG-70/30-I+P	BG-30/70-I+P
G60	10.87 ± 0.41 Cc	1.97 ± 0.12 Dc	18.21 ± 1.92 Bc	22.92 ± 0.52 Ab
I10	8.49 ± 0.74 Bd	3.26 ± 0.05 Bb	36.00 ± 3.88 Ab	40.66 ± 1.85 Aa
I60	17.33 ± 0.42 Cb	2.82 ± 0.09 Db	47.84 ± 4.72 Aa	38.54 ± 1.36 Ba
I90	19.59 ± 0.73 Ca	9.91 ± 0.62 Da	51.17 ± 1.88 Aa	37.83 ± 0.53 Ba

G60: 60 min gastric phase. I10, I60, I90: 10, 60 and 90 min intestinal phases, respectively. Mean values (*n* = 3) ± standard deviation. Different uppercase letters within rows indicate significant differences among formulations (*p* < 0.05). Different lowercase letters within columns indicate significant differences among digestion stages (*p* < 0.05). For sample denominations, see Table 1.

**Table 5:** FRAP (mg AAE/g BG) during simulated gastrointestinal digestion (GID) in BG formulations.

FRAP	BG-70/30-I	BG-30/70-I	BG-70/30-I+P	BG-30/70-I+P
G60	15.20 ± 0.81 Aa	2.36 ± 0.05 Bc	16.12 ± 0.38 Aa	4.88 ± 0.11 Bb
I10	6.98 ± 0.17 Bc	3.44 ± 0.17 Cb	11.38 ± 0.58 Ab	6.22 ± 0.44 Ba
I60	9.78 ± 0.27 Ab	3.53 ± 0.32 Ca,b	10.12 ± 0.21 Ac	6.28 ± 0.17 Ba
I90	9.97 ± 0.17 Ab	4.38 ± 0.58 Ca	10.26 ± 0.23 Ac	5.94 ± 0.44 Ba

G60: 60 min gastric phase. I10, I60, I90: 10, 60 and 90 min intestinal phases, respectively. Mean values (*n* = 3) ± standard deviation. Different uppercase letters within rows indicate significant differences among formulations (*p* < 0.05). Different lowercase letters within columns indicate significant differences among digestion stages (*p* < 0.05). For sample denominations, see Table 1.

**Table 6:** Fatty acid (FA) profile (mg FAME/g fresh BG) and sum of SFA, MUFA and PUFA in undigested BG formulations.

FA	BG-70/30-I; BG-70/30-I+P	BG-30/70-I; BG-30/70-I+P
C16:0	26.67 ± 0.02	71.86 ± 4.34
C17:0	0.206 ± 0.001	0.556 ± 0.035
C18:0	6.50 ± 0.01	17.55 ± 1.05
C20:0	1.11 ± 0.01	3.03 ± 0.18
C22:0	0.470 ± 0.001	1.26 ± 0.07
C23:0	0.069 ± 0.001	0.183 ± 0.003
C24:0	0.205 ± 0.001	0.567 ± 0.028
Σ SFA	35.23	95.00
C16:1n7	2.20 ± 0.02	5.93 ± 0.36
C18:1n7c	5.089 ± 0.007	13.80 ± 0.80
C18:1n9c	159.95 ± 0.22	430.91 ± 26.32
C20:1n9	0.702 ± 0.001	1.90 ± 0.11
Σ MUFA	167.94	452.54
C18:2n6c	22.97 ± 0.01	62.03 ± 3.77
C18:3n3	1.823 ± 0.002	4.91 ± 0.31
Σ PUFA	24.79	66.94

Mean values (*n* = 3) ± standard deviation. BG-70/30-I and BG-70/30-I+P, as well as BG-30/70-I and BG-30/70-I+P, exhibited identical FA profiles due to identical olive pomace oil (OPO) content. SFA, saturated fatty acids; MUFA, monounsaturated fatty acids; PUFA, polyunsaturated fatty acids. For sample denominations, see Table 1.

**Table 7:** Fatty acid (FA) profile (mg FAME/g soluble fraction, SF) and sum of SFA, MUFA and PUFA after gastrointestinal digestion (GID) of BG formulations.

FA	BG-70/30-I	BG-70/30-I+P	BG-30/70-I	BG-30/70-I+P
C16:0	7.54 ± 0.11	6.77 ± 0.07	31.23 ± 0.33	29.69 ± 0.43
C17:0	-	-	0.256 ± 0.001	0.239 ± 0.003
C18:0	1.85 ± 0.02	1.67 ± 0.03	7.95 ± 0.07	7.34 ± 0.10
C20:0	0.260 ± 0.001	0.214 ± 0.008	0.997 ± 0.021	0.975 ± 0.019
C22:0	-	-	0.462 ± 0.008	0.431 ± 0.019
C23:0	-	-	-	-
C24:0	-	-		-
**Σ SFA**	9.76	8.66	40.90	38.68
C16:1n7	1.22 ± 0.02	1.19 ± 0.01	3.26 ± 0.03	3.09 ± 0.06
C18:1n7c	2.65 ± 0.03	2.61 ± 0.03	7.46 ± 0.07	7.03 ± 0.02
C18:1n9c	77.78 ± 1.13	75.74 ± 0.49	221.26 ± 1.74	193.40 ± 8.86
C20:1n9	0.349 ± 0.006	0.340 ± 0.004	0.976 ± 0.007	0.849 ± 0.008
**Σ MUFA**	82.00	79.88	232.95	215.40
C18:2n6c	13.94 ± 0.18	13.44 ± 0.10	36.51 ± 0.32	34.80 ± 0.10
C18:3n3	0.928 ± 0.009	0.891 ± 0.006	2.43 ± 0.02	2.18 ± 0.03
**Σ PUFA**	14.92	14.33	38.94	36.98

Mean values (*n* = 3) ± standard deviation. SFA, saturated fatty acids; MUFA, monounsaturated fatty acids; PUFA, polyunsaturated fatty acids. For sample denominations, see Table 1.

**Table 8:** Bioaccessibility (BAC, %) of the main fatty acids (FAs) at the end of in vitro gastrointestinal digestion (GID) for the different BGs.

After In Vitro GID	BG-70/30-I	BG-30/70-I	BG-70/30-I+P	BG-30/70-I+P
**Main FAs**				
***SFA***				
Palmitic C16:0	28.28 ± 0.20 B	43.46 ± 2.51 A	25.40 ± 1.19 B	41.31 ± 2.15 A
Stearic C18:0	28.48 ± 0.31 C	45.31 ± 2.84 A	25.68 ± 1.06 C	41.85 ± 1.28 B
***MUFA***				
Vaccenic C18:1n-7	53.14 ± 0.94 A	54.03 ± 2.26 A	51.22 ± 2.06 A	50.97 ± 2.68 A
Oleic C18:1n-9	48.62 ± 1.05 A,B	51.35 ± 1.93 A	47.35 ± 1.97 B	44.88 ± 2.76 C
***PUFA***				
Linoleic C18:2n-6	60.68 ± 0.63 A	58.86 ± 2.70 A	58.51 ± 2.12 A	56.10 ± 2.94 A
Linolenic C18:3n-3	50.90 ± 0.60 A	51.20 ± 3.20 A	48.70 ± 0.60 A	46.80 ± 2.00 A

Mean values (*n* =3) ± standard deviation. SFA, saturated fatty acids; MUFA, monounsaturated fatty acids; PUFA, polyunsaturated fatty acids. Different uppercase letters within rows indicate significant differences among formulations (*p* < 0.05). For sample denomination, see Table 1.

**Table 9:** Pearson correlation coefficients (*n* = 12) between bioactive release, antioxidant activity and fatty acid (FA) bioaccessibility parameters at the end of intestinal digestion (I90).

	Inositols	Phenolics	ABTS	FRAP	Oleic	Linolenic	Total
**Inositols**	**1**	−0.730 **	−0.782 **	−0.215	0.913 ***	0.683 *	0.623 *
**Phenolics**		**1**	0.909 ***	0.172	−0.584 *	−0.526	−0.471
**ABTS**			**1**	0.508	−0.647 *	−0.537	−0.716 **
**FRAP**				**1**	−0.154	0.041	−0.711 **
**Oleic**					**1**	0.775 **	0.706 *
**Linolenic**						**1**	0.565
**Total**							**1**

Bioactive variables (inositols and phenolics) correspond to release values (%), and FA variables (oleic, linolenic and total) correspond to FA bioaccessibility (%). Significant correlations at *p* < 0.01 and *p* < 0.001 are highlighted in bold. * *p* < 0.05; ** *p* < 0.01; *** *p* < 0.001.

## Data Availability

All the raw data for this article, used in the generation of tables, are available at the public repository digital.csic.es of the Spanish Research Council (CSIC) at https://doi.org/10.20350/digitalCSIC/18477.
